# Assessment of muscle *Longissimus thoracis et lumborum* marbling by image analysis and relationships between meat quality parameters

**DOI:** 10.1371/journal.pone.0202535

**Published:** 2018-08-22

**Authors:** Elisa Giaretta, Attilio Luigi Mordenti, Giorgia Canestrari, Nico Brogna, Alberto Palmonari, Andrea Formigoni

**Affiliations:** Department of Veterinary Medical Sciences (DIMEVET), University of Bologna, Ozzano dell’Emilia, Bologna, Italy; University of Illinois, UNITED STATES

## Abstract

The intramuscular fat (IMF), recognized by the consumer as marbling, is an important meat quality trait. The objective of this study was to validate a new method of beef marbling evaluation by image analysis. The new assessment was compared with two known marbling measurements: chemical IMF and USDA scores. Moreover, the relationship between marbling measurements and other meat quality parameters was investigated. Samples of *Longissimus thoracis et lumborum* (LTL) muscle were obtained from carcasses of 39 Italian crossbred heifers and 62 Angus bred heifers, that underwent two different dietary treatments. The use of various breeds and diets was chosen to obtain different intramuscular fat levels, in order to validate the use of Image J software for the analysis of different type of beef meat. The images of fresh cuts were appraised by experienced beef graders, and the samples were used to determine fat content by chemical Soxhlet extraction. Carcasses measurements according to the EUROP system, and other physical meat proprieties were also assessed. The results demonstrated that the marbling measurements obtained by computer image analysis, such as the number of marbling particles, the average particle size (mm), and the percentage of marbling particles (%), significantly (P < 0.05) correlated with USDA scores and IMF content. Moreover, the principal component analysis (PCA) showed three principal meat components, identified as 1) color, 2) fat, and 3) water release. The second principal component (PC) explained 24.94% of variance, and was positively correlated with image analysis measures, USDA score, and IMF, while negatively correlated with the Warner–Bratzler shear force (WBSF).

## Introduction

Marbling is an important measure for many beef quality grading systems. It affects taste, juiciness, tenderness and flavor, and acts as a key factor for consumers when purchase meat products [1–4]. In European Union (EU) countries beef carcasses are evaluated under the EUROP system. This system describes the carcass conformation (E.U.R.O.P) and external fatness (1–5), but does not report marbling indicators. Moreover, the EUROP system is not designed to assess the quality of individual cut of beef meat. Others major existing grading systems, such as the American, Australian, and Japanese one, consider marbling or intramuscular fat (IMF) content as one of the main factor to assess beef meat quality [5]. In both United States Department of Agriculture (USDA) and Australian Meat Standards (MSA) systems, marbling is evaluated on a single cross-section of the *Longissimus thoracis et lumborum* (LTL) muscle, which would provide a whole carcass estimate [6]. According to the USDA system, degree of marbling is the primary determination of meat carcass grade, and each grade of marbling is divided into 100 subunits [7,8], from “abundant” to a “practically devoid” grade. Thus, it is extremely important to have a reliable and adequate analytical measurement. In order to objectivize marbling measures, image analysis methods have been developed using different imaging software [9–15]. According to these methods, the rib eye color images were binarized and converted in black/white images, allowing both the separation of meat from intramuscular fat, and the evaluation of fat area ratio.

Image J computer software (https://imagej.nih.gov/ij/), used for our investigation, is an open source image processing program designed for scientific multidimensional images and it has already been used for the prediction of IMF from ultrasonographic images [14].

Several works have already used the marbling measures obtained by image analysis to predict chemical IMF [11–14]. However, to our knowledge, there are no study have been done to correlate analysis obtained from open source Image J software, USDA scores and IMF.

The aim of our study was to investigate the ability of Image J software to assess muscular marbling. For this purpose, LTL muscles with different level of IMF, within two groups of carcasses (HIGH FAT and LOW FAT group) were chosen for the study. The correlations between alternative marbling measurements approaches (chemical IMF and USDA scores) were used to validate the new assessment. In addition, the relationships of marbling measurements with the other meat quality parameters were investigated.

## Material and methods

The Scientific Ethic Committee on Animal Experimentation of the University of Bologna examined and approved the experimental protocol (n.: 4783-X/10 All: 17) used in this study.

### Samples collection and treatment

Samples of LTL muscle were obtained from carcasses of 39 Italian crossbred heifers and 62 Angus bred heifers, underwent two different dietary treatment in order to obtain different amount of intramuscular and carcass fat. Thus, the two experimental groups were called LOW-FAT and HIGH-FAT groups, respectively. Meat samples of the two different groups were used to verify if the image J software could be able to identify various grades of marbling.

Cattles were fed once per day at 07:30 in the morning and received *ad libitum* diet and free access to fresh water. Diets were prepared as total mixed rations (TMR) with a horizontal mixer wagon (Zago 13-m^3^, ZAGO srl, PD, Italy) equipped with a weighing scale. The LOW-FAT group received a hay-based diet with high percentage of roughage, composed by alfalfa hay (28.5%) and grass hay (28.5%). The diet included earlage (28.5%), wheat hulls (9.5%), and a protein premix (5%). The HIGH-FAT group received a diet composed by corn silage (41%), earlage (24%), wheat hulls (10%), corn meal (9%), wheat straw (7%) protein supplement (5%) and flaxseed (4%). The two diets differed in forage content (57.14% vs 48.28% on dry matter basis, for LOW-FAT and HIGH-FAT group, respectively), and lipids (2.73% vs 3.51% on DM, respectively). Each box contained 8 animals (4.5 m^2^ space per head). Animals were slaughtered at an average body weight (SBW) of 552 ± 71 kg, following the standard weight slaughtering procedures. Process was completed at the local abattoir in the Department of Veterinary Medical Science of Bologna (DIMEVET).

After slaughtering, the carcasses were graded for degree of fatness (on a scale of 1–5. with 5 being the highest degree of fatness), and conformation according to the E.U.R.O.P. rank (Table 1). The letters were converted into integers for better statistical analysis, and each class was subdivided into 3 to give 15 subclasses: 1 corresponded to P− (the worst) and 15 to E+ (the best) according to the European beef grading system [16]. Then, the hot carcass weight (HCW) was measured. The carcass yield (%) was calculated as the ratio between HCW and SBW.

**Table 1:** Carcass counts for categorical variables.

**EUROP conformation**^a^	**E-**	**E**	**E+**	**U-**	**U**	**U+**	**R-**	**R**	**R+**	**O-**	**O**	**O+**	**P-**	**P**	**P+**
LOW FAT	−	−	−	−	18	−	−	32	−	−	−	−	−	−	−
HIGH FAT	−	−	−	−	23	−	−	27	−	−	−	−	−	−	−
**EUROP fatness**^b^	**1-**	**1**	**1+**	**2-**	**2**	**2+**	**3-**	**3**	**3+**	**4-**	**4**	**4+**	**5-**	**5**	**5+**
LOW FAT	−	−	−	−	34	−	−	8	−	−		−	−		8
HIGH FAT	−	−	−	−	2	−	−	3	−	−	1	−	−	20	24

^a^ The EUROP conformation scores are ranked from “E” (highest muscularity) to “P” (poorest), and could be subdivided into + or–to create 15 point description.

^b^ The EUROP fatness system utilizes 5 fat classes, with “1” being the leanest and “5” the fattest. These five classes could be further subdivided into–or + designations to provide a 15 point scale.

Carcasses were stored at 2–4 °C in the local abattoir immediately after the slaughtering procedures, and samples of LTL muscle from the 13^th^ rib were collected at 72 h postmortem to determine the meat quality characteristics and composition. Samples were placed in air-tight plastic bags and transported on ice to the laboratory for the subsequent analysis. Briefly, each LTL cut was photographed for the successive marbling assessment, and USDA marbling score for each meat sample was recorded. A cube of 5x5x5 cm of approximately 80–100 g (± 0.05 g) was cut from each sample and immediately evaluated for color analysis. The color of each sample was assessed according to the CIE Lab (L*, a*, b*) color space [17], using a Minolta Chromameter CR-200 (Minolta Camera Co. Ltd. Osaka. Japan) equipped with a D65 illuminant. Readings for each of L*, a*, b* values (L* = lightness—range: 0 = black to 100 = white; a* = red-green shift—range: -50 = green to +50 = red; b* = yellow-blue shift—range: -50 = blue to +50 = yellow), standardized against a white tile (L* = 97.78. a* = 0.19. b* = 1.84), were taken at two spots on the side of the meat sample exposed to the light meat color. Hue and chroma (c*) were calculated as follows: Hue = arctan (b*/a*); c* = (a*2+b*2)1\2 [18] Later, the same meat sample was used for drip loss (DL), cooking loss (CL) and tenderness analysis. Drip loss and cooking loss were evaluated in samples taken from the LTL muscle according to the method described by [19]. For DL determination, the meat sample was placed into a plastic box over a supporting mesh (ensuring no contact between sample and box) and then sealed. After 24 h at cold temperature (1–5 °C) the samples were taken out from the box dabbed lightly on filter paper and weighed again. Drip loss was expressed as a percentage of the initial weight (weight 1). For CL determination, each sample previously used for DL was weighed (weight 2) and placed in a thin plastic bag with opening above the surface. Sample was left in a boiling–water bath until the core temperature reached 75°C. The temperature was continuously monitored by a thermometer with a thermocouple (Eurotron Micrologger 2) inserted into each sample. Once the temperature was reached, samples were cooked for additional 15 minutes, then pulled out from the water bath, cooled under tap water for 20 min, and stored at 4°C until equilibrated. The meat was collected from the bag, dabbed lightly on filter paper and weighed (weight 3). CL was expressed as a percentage of the initial sample weight according to the following equation: cooking loss = 100x(weight 3 -weight 2)/weight 2). Warner–Bratzler shear force (WBSF) for meat tenderness evaluation was determined by AMSA methods [20]. Cooked meat samples were cooled for 24h at 4°C. Seven round cores (1.27 cm diameter) were removed from each sample parallel to the long axis of the muscle fibers by a mechanical coring device. Each core was sheared once through the center by a WBSF device (V-shape blade) connected to an Instron Universal Testing Machine (Model 1011. Instron Corp. Canton. MA). A 50-kg compression load cell and 200 mm/min cross head speed were used. WBSF was expressed as peak force (N). The seven cores were averaged for statistical analysis.

About 100 g of LTL muscle was frozen at -20°C and subsequently lyophilized and grinded to obtain homogeneous and moisture-free samples used for the chemical analysis. The measurement of intramuscular fat (IMF) content was determined by the Soxhlet method (Soxtec System HT6. Tecator AB) on lyophilized samples [21].

### Marbling analysis

**Fig 1:**
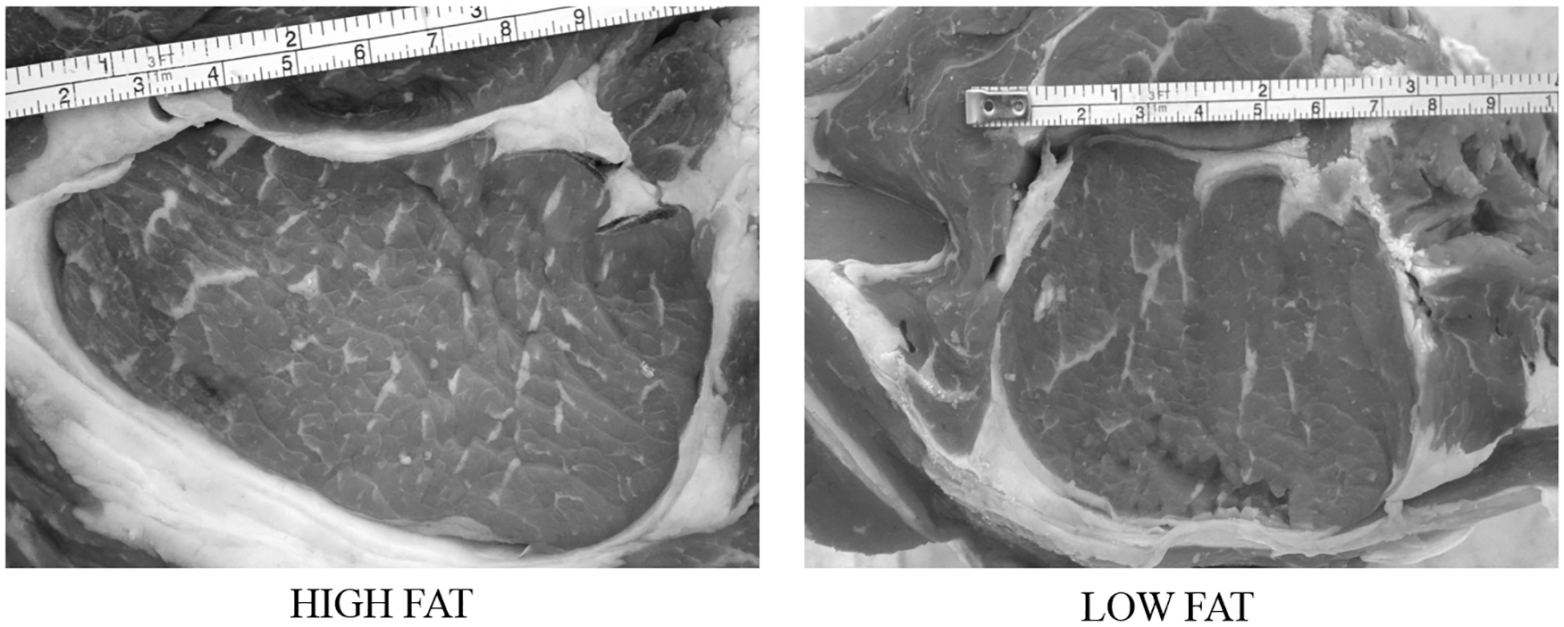
Sample images acquired by a digital camera system.

**Fig 2:**
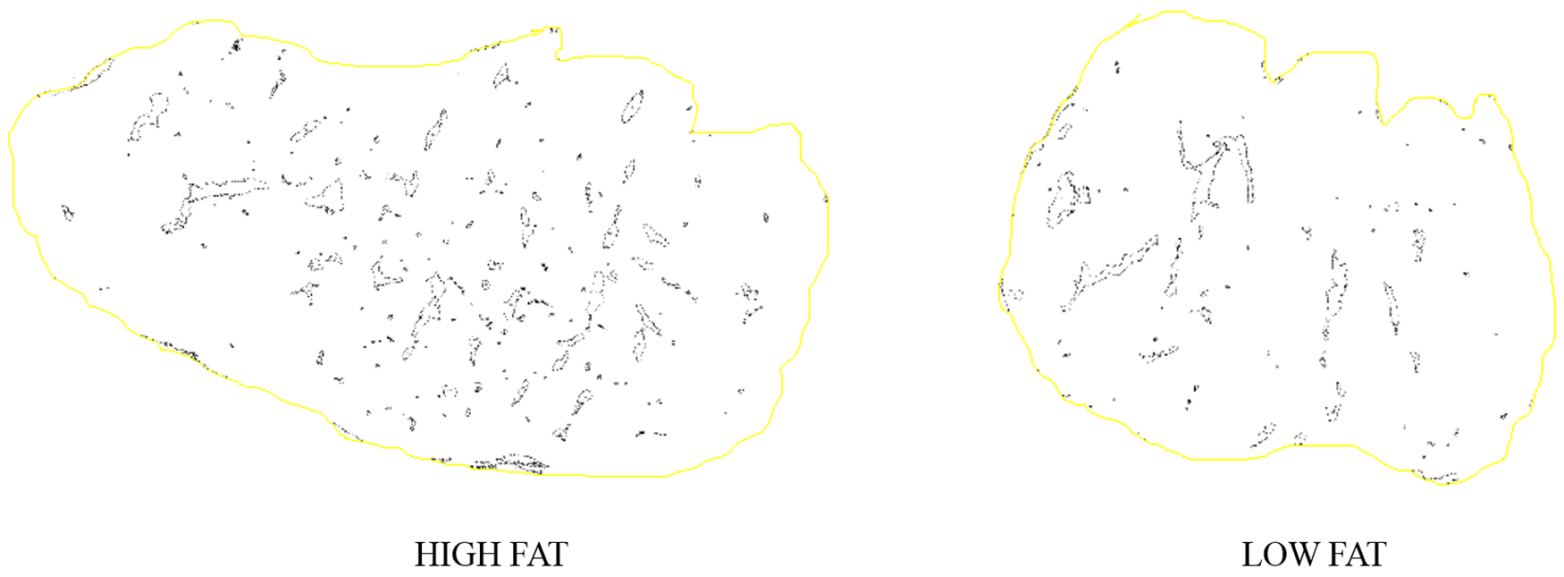
Image analysis of LTL samples with Image J software.

The meat samples were photographed by a digital camera system: CANON EOS 1000D camera mounted on a jig at 40 cm height, with a manual setting of ISO 1600, shutter speed 1/13 and f-number 4.5. Each sample was photographed over a blank surface and a ruler was put over the meat cut to obtain the pixel: mm ratio for the successively image analysis (Figs 1 and 2).

To evaluate marbling characteristics the photographic image was analyzed with Image J software. The determination of the marbling characteristics was performed after several steps. The first step was to set the scale pixel/mm, converting pixels to mm measurement units, through the ruler present in the photographic image. Then each image was converted into grey scale 8-bit image, and the threshold was adjusted to highlight the marbling fat over the background. The part of the image containing the LTL was manually selected before the analysis of particles was performed. The minimum size of the fat particles considered as marbling fleck was set at 1mm. From the image analysis the following characteristics were determined: number of marbling particles, average particle size (mm), and percentage of marbling particles (%). The percentage of marbling area was calculated as the marbling particles area related to the image selected area, expressed as percentage. To eliminate subjective operator differences, image acquisition and measurements were done by a single experienced operator.

The individual images, saved as JPEG files, were subsequently independently evaluated by highly experienced grader, who assigned marbling scores in accordance to the USDA system [22]. In USDA assessment, marbling was assigned by graders in 10 point increments within a scale of 100 [8].

### Statistical analysis

Analysis of results was conducted using a statistical package (IBM^®^ SPSS^®^ 21.0 for Windows; IBM Corp.; Chicago, IL, USA). The level of significance was set at *P* ≤ 0.05 in all cases. Correlations between variables were analyzed by Pearson and Spearman tests. When missing data were encountered, correlations were estimated on a pairwise complete basis, i.e. the set of complete cases were analyzed.

A Principal Component Analysis (PCA) was performed to reduce the variable numbers and to identify any latent structures in the data. Briefly, the quality parameters obtained for each meat sample (L*, a*, b*, hue, chroma, DL, CL, WBSF, IMF, USDA score, percentage of marbling area, number of marbling particles and average particle size) were used as independent variables in a PCA. Prior to perform the PCA, the Kaiser-Mayer-Olkin (KMO) measure of sample adequacy and the Bartlett test of sphericity were conducted. Variables were standardized using Z-scores and sorted into PCA components. The obtained data matrix was rotated using the Varimax procedure with the Kaiser normalization. As a result, regression scores for each of the PCA components were calculated per meat sample, and a new variable for each PCA component was created. Results are expressed as mean percentage ± standard deviation.

## Results and discussion

### Carcass measurements

As shown in Table 2 there was a significant and strong correlation between SBW and HCW (r = 0.97, *P* < 0.01). A significant correlation was observed among carcass yield and the EUROP conformation scores (r = 0.36, *P* < 0.01). Moreover, the EUROP fatness score was significantly correlated with IMF, SBW, and HCW (IMF: r = 0.34, *P* < 0.01; SBW: r = 0.53, *P* < 0.01; HCW: r = 0.52, *P* < 0.01). No significant correlations were highlighted between carcass measurements and IMF, nor between carcass measures and the marbling measures (USDA and Image J scores).

**Table 2:** Correlations between meat quality characteristics.

	SBW	HCW	Yeld	Fatness	EUROP	IMF	USDA	DL	CL	WBSF	L*	a*	b*	Hue	c*	marbling particles (n)	Average particle size	Marbling percentage
SBW	1.00	0.97°°	0.12	0.53°°	0.14	-0.01	0.02	-0.28	-0.07	-0.05	0.13	0.30°°	0.25°	-0.02	0.29°°	0.15	-0.09	0.01
HCW	0.97°°	1.00	0.27°°	0.52°°	0.20	-0.02	0.05	-0.30	-0.13	-0.03	0.16	0.32°°	0.26°	-0.04	0.30°°	0.14	-0.06	0.03
Yeld	0.12	0.27°°	1.00	0.04	0.36°°	-0.07	0.11	-0.01	-0.17	0.03	0.07	0.04	0.02	-0.01	0.04	-0.10	-0.06	-0.03
Fatness	0.53°°	0.52°°	0.04	1.00	0.19	0.34°°	0.27°°	-0.13	-0.13	-0.08	-0.10	0.06	0.02	-0.11	0.05	0.27°	0.05	0.30°°
EUROP	0.14	0.20	0.36°°	0.19	1.00	0.15	0.12	-0.07	-0.04	-0.03	-0.14	-0.01	-0.06	-0.08	-0.02	0.02	-0.10	-0.02
IMF	-0.01	-0.02	-0.07	0.34°°	0.15	1.00	0.56°°	-0.15	-0.12	-0.29°	-0.19	-0.26	-0.20	0.08	-0.28	0.39°°	0.26°	0.62°°
USDA	0.02	0.05	0.11	0.27°°	0.12	0.56°°	1.00	-0.20	-0.27	-0.26°	-0.03	0.01	0.05	0.08	0.02	0.57°°	0.56°°	0.83°°
DL	-0.30	-0.30	-0.01	-0.13	-0.07	-0.15	-0.20	1.00	0.12	-0.12	-0.40	-0.46	-0.40	0.02	-0.44	0.11	-0.01	-0.14
CL	-0.07	-0.13	-0.17	-0.13	-0.04	-0.12	0-.27°	0.12	1.00	-0.03	0.00	0.01	0.03	0.21	0.02	-0.28	-0.02	-0.26
WBSF	-0.05	-0.03	0.03	-0.08	-0.03	-0.29°	-0.26°	-0.12	-0.03	1.00	0.05	0.14	0.09	-0.11	0.12	-0.19	-0.15	-0.29°
L*	0.13	0.16	0.07	-0.10	-0.14	-0.19	-0.03	-0.40	0.00	0.05	1.00	0.78°°	0.87°°	0.56°°	0.82°°	-0.14	0.07	-0.02
a*	0.30°°	0.32°°	0.04	0.06	-0.01	-0.26	0.01	-0.50	0.01	0.14	0.78°°	1.00	0.95°°	0.27°°	0.99°°	-0.14	0.10	-0.07
b*	0.25°	0.26°	0.02	0.02	-0.06	-0.20	0.05	-0.40	0.03	0.09	0.87°°	0.95°°	1.00	0.52°°	0.98°°	-0.11	0.12	-0.01
Hue	-0.02	-0.04	-0.01	-0.11	-0.08	0.08	0.08	0.02	0.21	-0.11	0.56°°	0.27°°	0.52°°	1.00	0.35°°	0.00	0.15	0.11
c*	0.29°°	0.30°°	0.04	0.05	-0.02	-0.28	0.02	-0.44	0.02	0.12	0.08°°	0.99°°	0.98°°	0.35°°	1.00	-0.13	0.10	-0.06
marbling particles (n)	0.15	0.14	-0.10	0.27°	0.02	0.39°°	0.57°°	0.11	-0.28	-0.19	-0.14	-0.14	-0.11	0.00	-0.13	1.00	0.23°	0.71°°
Avarage particle size	-0.09	-0.06	-0.06	0.05	-0.10	0.26°	0.56°°	-0.01	-0.02	-0.15	0.07	0.10	0.12	0.15	0.10	0.23°	1.00	0.58°°
Marbling percentage	0.01	0.03	-0.03	0.30°°	-0.02	0.62°°	0.83°°	-0.14	-0.26	-0.29°	-0.02	-0.07	-0.01	0.11	-0.06	0.71°°	0.58°°	1.00

SBW, slaughter body weight; HCW, hot carcass weight, Yield, carcass yield, Fatness, Europ fatness score, EUROP, Europe carcass conformation; IMF, chemical intramuscular fat; USDA, USDA marbling score, DL, drip loss; CL, cooking loss; WBSF, Warner–Bratzler shear force. L*, a*, b*; Hue, C* are color parameters; number of marbling particles, average particle size and the marbling percentage are the marbling characterization by Image J software.

° indicates that the correlation (r) is significantly different to zero at the 0.05% of significance.

°° indicates that the correlation (r) is significantly different to zero at the 0.01% of significance.

While the correlations between carcass measures and carcass age were obvious and already well known [23,24], it was of our interest to determine the strength of any relationship between primary carcass fat and marbling measures. These results are in agreement with [25] and [6] studies, which raised questions about the ability of EUROP carcass measures to accurately predict marbling and fat composition of cuts.

### Meat quality characteristics

The correlations between meat quality characteristics are shown in Table 2. A significant negative correlation (*P* < 0.01) was observed between meat color measures and drip loss (L*: r = −0.40; a*: r = −0.46; b*: r = −0.40). These results are difficult to interpret, whereas both values are related to water holding capacity, and higher color parameters are usually linked to excessive water losses [26]. In addition, no significant correlations were found among drip, cooking loss and tenderness of cooked meat.

### Intramuscular fat scoring using image analysis

The marbling measures were obtained by an automatic image analysis with Image J and a visual evaluation with the USDA system. The output of Image J analysis software provided a range of measures including the number of marbling particles, the average size of marbling particles, and the marbling percentage derived from the relative white versus red area in total (%). The LOW-FAT and HIGH-FAT group composition for chemical IMF and marbling measures is reported in Table 3. The descriptive statistic measured a higher chemical IMF in the HIGH-FAT group than in the LOW-FAT one. In addition, the percentage of marbling assessed with Image J and USDA system resulted numerically higher in HIGH-FAT than in LOW-FAT group, for both evaluation methods. The marbling particle value was also observed numerically higher in HIGH FAT group.

**Table 3:** Means of image analysis marbling measures and IMF of Longissimus thoracis of LOW FAT and HIGH FAT groups.

	LOW-FAT	SD	HIGH-FAT	SD
Chemical intramuscular fat (% as it is)	5.17	1.91	6.66	1.95
Marbling percentage	6.12	2.96	8.26	3.56
Average particle size (mm)	5.33	1.44	5.26	1.63
Number of marbling particles	40.71	18.37	50.41	20.38
USDA marbling score	474.87	79.3	522.1	87.41

**Fig 3:**
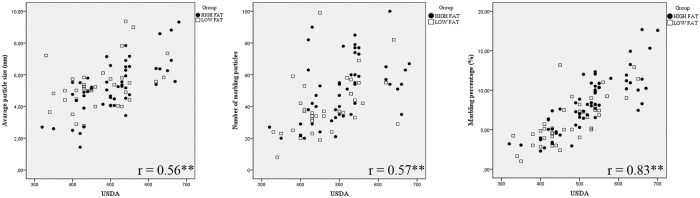
Scatter plots of USDA marbling score against the Image J marbling measures of LTL muscle. The presence of a * indicates that the correlation (r) is significantly different to zero at the 0.05% of significance. The presence of two ** indicates that the Pearson correlation (r) is significantly different to zero at the 0.01% of significance.

**Fig 4:**
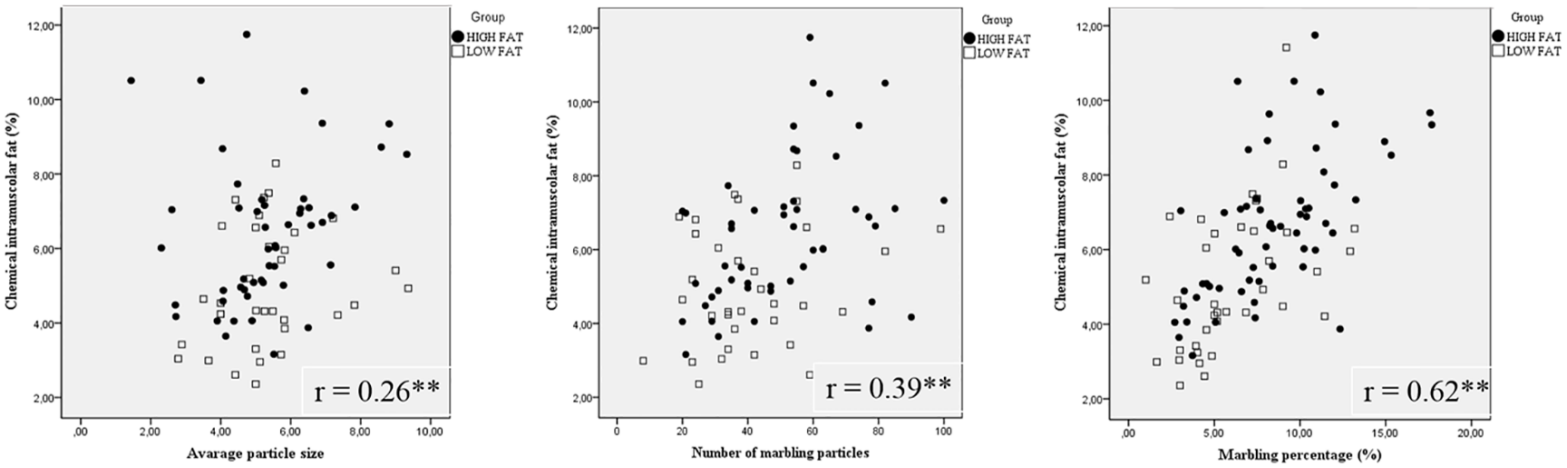
Scatter plots of chemical intramuscular fat (IMF) against the Image J marbling measures of LTL muscle. The presence of a * indicates that the correlation (r) is significantly different to zero at the 0.05% of significance. The presence of two ** indicates that the Pearson correlation (R) is significantly different to zero at the 0.01% of significance.

Significant (*P* < 0.01) and interesting correlations were observed between Image J marbling measures and USDA score system (Fig 3). The stronger correlation (r = 0.83) was found between USDA scores and Image J marbling percentages, suggesting that the image analysis would be an appropriate assessment of intramuscular marbling. Moreover, significant correlations (*P* < 0.01) were observed among USDA and the other Image J marbling measures (number of marbling particles: r = 0.57; average particle size: r = 0.56). To confirm this result, significant (*P* < 0.01) correlation between Image J marbling measures (except for the average particle size) and chemical IMF were observed (number of marbling particles: r = 0.39; marbling percentage: r = 0.62, Fig 4). Moreover, chemical IMF was more correlated (*P* < 0.01) to the marbling percentage measured by Image J analysis, rather than USDA marbling score (Image J: r = 0.62; USDA: r = 0.56). These results are in agreement with those obtained by [14], who used the same software (Image J) to analyze ultrasound images. In this investigation, the authors observed that the best predictors of chemical IMF were the proportion of marbling particles (marbling percentage) and the marbling particle area, while the number of marbling particles had a weak linear relationship with IMF. Significant correlations between the chemical IMF and the percentage of marbling particles area were also observed by [11,27].

Finally, in our study marbling percentage (Image J), USDA score, and chemical IMF were negatively (*P* < 0.05) correlated with WBSF of cooked meat (Image J: r = −0.29; USDA: r = −0.26; IMF: r = −0.29). Although these variables were poorly correlated, their negative relationship is already known, as well as the consequent positive correlation between meat tenderness and fat content [28,29]

Considered together, these results allowed us to assume that marbling Image J assessment could be routinely used for intramuscular marbling evaluation, and it would be a very good indicator of meat intramuscular fat and meat quality in general. Moreover, this tool can objectively detect more marbling features than the routinely visual assessments, such as particles number and size.

### Principal component analysis

The principal component analysis, after Varimax rotation, resulted in three principal components, which together account for 69.55% of variance. As shown in Table 4, the first PC explained 34.70% of variance and it was positively correlated with L*, a*, b*, while negatively correlated with hue and DL. The second PC explained 24.94% of variance, and it was positively correlate with number of marbling particles, average particle size (mm), percentage of marbling area, USDA score, and IMF. Moreover, the second PC was negatively correlated with WBSF. The third variable explained 9.91% of variance, and it was positively correlated with CL and DL, and negatively correlate with hue and WBSF.

**Table 4:** Principal components loadings and variance.

	Principal Components		
	1	2	3
Number of marbling particles		0.67	
Average particle size (mm)		0.66	
Marbling percentage (%)		0.95	
USDA marbling score		0.88	
Intramuscular fat (%)		0.75	
WBSF (N)		-0.38	-0.40
L*	0.91		
a*	0.96		
b*	0.98		
Hue	-0.48		-0.66
c*	0.97		
Drip loss (%)	-0.64		0.45
Cooking loss (%)			0.64
Proportion of variance	34.70	24.94	9.91
Cumulative variance	34.70	59.64	69.55

Principal component analysis’ report of component loadings, variances and description of the different impact of each single component (proportion of variance and cumulative variance).

**Fig 5:**
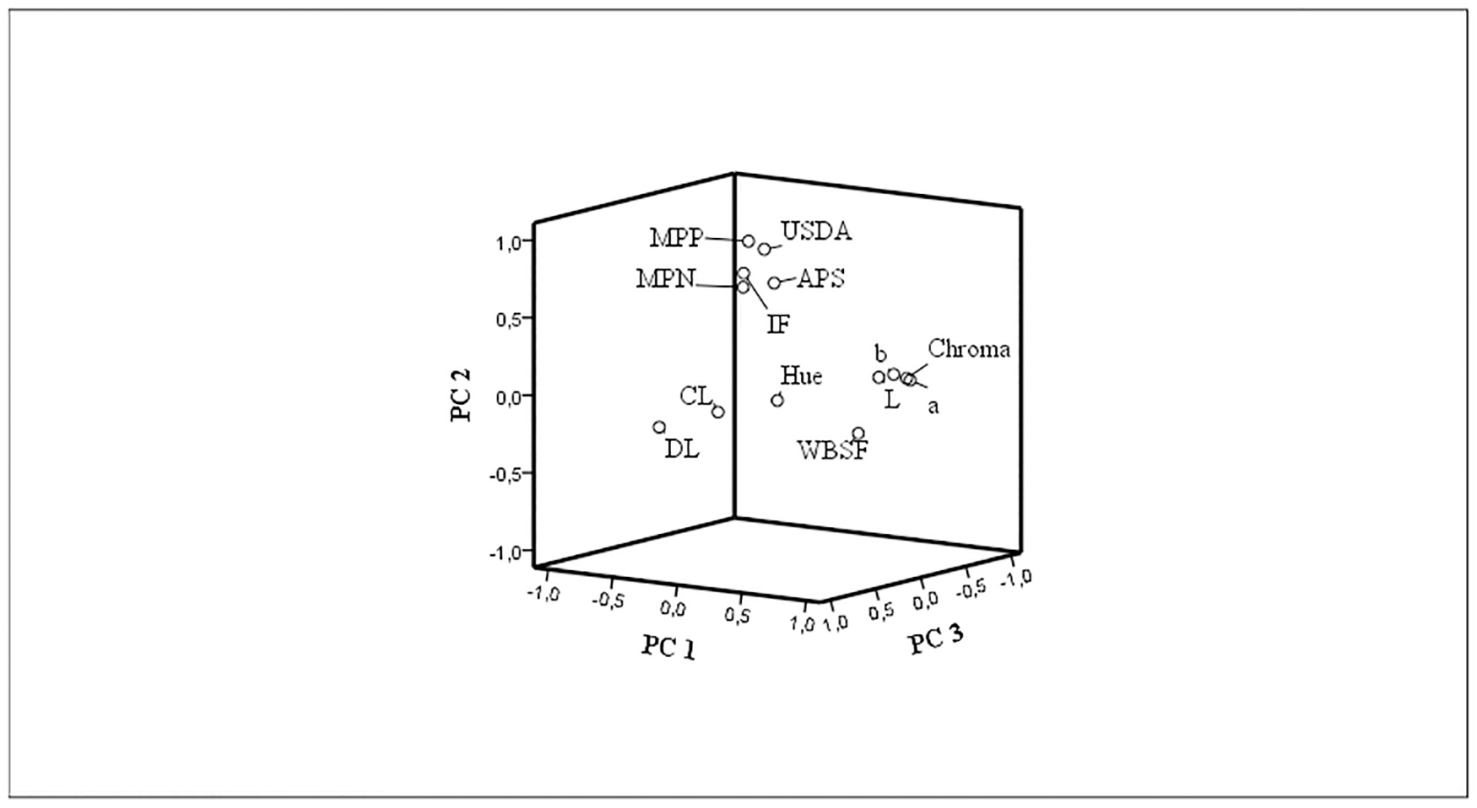
Plots of the first three PC loading vectors. Drip loss (DL); cooking loss (CL); lightness (L); red-green shift (a); yellow-blue shift (b); hue; chroma (c); Warner–Bratzler shear force (WBSF); marbling particle percentage (MPP); marbling particle number (MPN); average particle size (APS); USDA marbling score (USDA); intramuscular fat (IMF).

Thus, as shown in the loading plot (Fig 5), the first two PCs highlighted two distinct groups of variables, which we identify as: 1) meat fat; 2) meat colour. The third PC, which is positively correlated with CL and DL, and negatively with WBSF and hue, could be identified as meat water release.

## Conclusions

It was concluded that the computer image analysis of meat marbling was a reliable and objective approach to measure meat fat content, both in fatty and in lean meats. The percentage of marbling particles obtained by image analysis, was significantly correlated with USDA marbling scores (r = 0.83) and chemical IMF (r = 0.61). Moreover, the image analysis allowed the identification of other marbling features, such as the marbling particle size and the number of marbling particles.

Finally, the second PC from Principal Component Analysis was positively correlated with marbling Image J measures, USDA scores and IMF, suggesting that marbling image analysis could be a valid alternative to measure the intramuscular fat.

In conclusion, the use of a computer image analysis could be a convincing and non-destructive approach for marbling assessment, and it could be implemented into the existing meat quality grading systems.

## Supporting information

S1 Dataset Correlations. (XLS)
